# *In silico* analysis of the V66M variant of human BDNF in psychiatric disorders: An approach to precision medicine

**DOI:** 10.1371/journal.pone.0215508

**Published:** 2019-04-18

**Authors:** Clara Carolina Silva De Oliveira, Gabriel Rodrigues Coutinho Pereira, Jamile Yvis Santos De Alcantara, Deborah Antunes, Ernesto Raul Caffarena, Joelma Freire De Mesquita

**Affiliations:** 1 Department of Genetics and Molecular Biology, Bioinformatics and Computational Biology Laboratory, Federal University of the State of Rio de Janeiro (UNIRIO), Rio de Janeiro, Rio de Janeiro, Brazil; 2 Computational Biophysics and Molecular Modeling Group, Scientific Computing Program (PROCC), Fundação Oswaldo Cruz, Manguinhos, Rio de Janeiro, Brazil; UMR-S1134, INSERM, Université Paris Diderot, INTS, FRANCE

## Abstract

Brain-derived neurotrophic factor (BDNF) plays an important role in neurogenesis and synapse formation. The V66M is the most prevalent BDNF mutation in humans and impairs the function and distribution of BDNF. This mutation is related to several psychiatric disorders. The pro-region of BDNF, particularly position 66 and its adjacent residues, are determinant for the intracellular sorting and activity-dependent secretion of BDNF. However, it has not yet been fully elucidated. The present study aims to analyze the effects of the V66M mutation on BDNF structure and function. Here, we applied nine algorithms, including SIFT and PolyPhen-2, for functional and stability prediction of the V66M mutation. The complete theoretical model of BNDF was generated by Rosetta and validated by PROCHECK, RAMPAGE, ProSa, QMEAN and Verify-3D algorithms. Structural alignment was performed using TM-align. Phylogenetic analysis was performed using the ConSurf server. Molecular dynamics (MD) simulations were performed and analyzed using the GROMACS 2018.2 package. The V66M mutation was predicted as deleterious by PolyPhen-2 and SIFT in addition to being predicted as destabilizing by I-Mutant. According to SNPeffect, the V66M mutation does not affect protein aggregation, amyloid propensity, and chaperone binding. The complete theoretical structure of BDNF proved to be a reliable model. Phylogenetic analysis indicated that the V66M mutation of BDNF occurs at a non-conserved position of the protein. MD analyses indicated that the V66M mutation does not affect the BDNF flexibility and surface-to-volume ratio, but affects the BDNF essential motions, hydrogen-bonding and secondary structure particularly at its pre and pro-domain, which are crucial for its activity and distribution. Thus, considering that these parameters are determinant for protein interactions and, consequently, protein function; the alterations observed throughout the MD analyses may be related to the functional impairment of BDNF upon V66M mutation, as well as its involvement in psychiatric disorders.

## Introduction

Psychiatric disorders are polygenic and multifactorial brain syndromes [1] characterized by clinically significant behavioral, psychological or biological dysfunction [2]. Psychiatric disorders are considered a major public health problem and are responsible for severe distress and functional impairment in individuals with notorious consequences in those who have been affected and their families and social and work environments [3]. Although the global burden of psychiatric disorders is mainly assigned to the disability and not to the mortality of affected individuals [4], mortality rates in psychiatric patients are much higher than those reported in the general population [5,6]. It is estimated that more than 50% of the population in middle and high-income countries will suffer from at least one psychiatric disorder throughout life. In addition, the estimated cost of these diseases between 2011 and 2030 is thereby projected at US$ 16.3 trillion dollars, which makes the economic cost of psychiatric disorders comparable to that of cardiovascular diseases and higher than that of cancer, diabetes and chronic respiratory diseases [3].

Although there is no consensus on the cause of psychiatric disorders [1,7], most studies suggest they are caused by neurotransmitters deregulation, genetic abnormalities, and defects in the structure or function of the brain [7]. In this context, non-synonymous mutations in the *BDNF* gene are implicated in the development of psychiatric disorders because they affect the function and distribution of brain-derived neurotrophic factor (BDNF), which is crucial for central nervous system development, neurogenesis and neuronal plasticity [8].

BDNF is a 247-amino acid secretory protein encoded by the *BDNF* gene [9], which is located on chromosome *11p14*.*1* [10]. BDNF is synthesized as a precursor protein, pre-pro-BDNF, which is cleaved into pro-BDNF in the endoplasmic reticulum (ER). Pro-BDNF is addressed and might be further cleaved into mature BDNF (m-BDNF) inside vesicles in the trans-Golgi network. Both dimeric pro-BDNF or dimeric m-BDNF are secreted [8] through activity-dependent exocytosis in excitable cells secreting granules or through constitutive release in all cell types [11]. The secreted homodimers bind to their cognate receptor tropomyosin kinase (Trk) and p75 neurotrophin receptor [12], inducing a series of intracellular signaling cascades related to proliferation, maturation, and maintenance of the neuronal function [8,13].

The most frequent missense mutation of BDNF in humans occurs in the pro-region of the protein [10] and comprises the substitution of a valine to a methionine at position 66 (V66M) [13]. The pro-region of BDNF, particularly the position 66 and its adjacent residues, are determinant for the intracellular sorting and activity-dependent secretion of this molecule [14]. The V66M mutation is believed to impair protein function and distribution [14,15] by interrupting folding, dimerization and intracellular trafficking of the protein [16], in addition to preventing the release-dependent activation of BDNF [14]. This mutation is also associated with the development of bipolar disorder, depression, obsessive-compulsive disorder, eating disorders, schizophrenia [15], anxiety, addiction, and memory and learning disabilities [13]. The structure of the residues 136 to 244 of BDNF have been solved by X-ray crystallography (PDB ID: 1BND and 1B8M) [17]. However, there is no experimentally determined structure for the V66M mutation and its adjacent residues [17].

Knowledge of three-dimensional structures is essential for understanding the mechanisms by which proteins perform their functions [18] and how these mechanisms can be disrupted by disease-associated mutations [19]. However, the determination of protein structures by conventional methods, such as X-ray diffraction and nuclear magnetic resonance (NMR), is expensive and time-consuming [20], increasing the imbalance between the amount of structures elucidated by experimental methods and the number of protein sequences [18] determined by rapid, massively parallel and low-cost sequencing methods [19]. In this scenario, computational (*in silico*) approaches, are efficient [20] and necessary to predict protein structures [18] and study disease-associated mutations [19] because they enable modeling of three-dimensional structures and prediction of the effect of mutations on protein function based on the amino acid sequence, in a cheaper, faster, and more accurate way when compared to experimental methods [18,20].

In addition to contributing to the development of pathologies [21], missense mutations can also influence the treatment regimen adopted. Understanding the effects of such mutations on the structure and function of a protein is important for precision medicine and the development of new drugs [22]. The characterization of biochemical and structural parameters constitutes the main approach used to understand the functional effects of missense mutations [19]. Despite the central role of BDNF in psychiatric disorders development, little is known about the structural and functional effects of its mutations.

In this work, we used the computational methodology previously described by our group [20,23–25] to analyze the structural and functional effects of the V66M variant of BDNF, which has been associated with several psychiatric disorders [13].

## Materials and methods

### Dataset

Sequences and information about BDNF and its natural variant V66M were retrieved from the UniProt [9] (UniProt ID: P23560), OMIM (OMIN ID: 113505) [26] and PubMed [10] databases. The BDNF crystallographic structure with the highest resolution on the Protein Data Bank (PDB) database [17] (PDB ID: 1BND) was selected as the modeling template [27].

### Functional and stability prediction analysis

The functional and stability effects of the V66M mutation were predicted using the following algorithms: PhD-SNP [28], PMut [29], PolyPhen-2 [30], SIFT [31], SNAP [32], SNPS&GO [33], VarMod [34], SNPeffect4.0 [22] and I-Mutant2.0 [35].

### Structural modeling

Complete three-dimensional structures of BDNF were created using *ab initio* and comparative modeling strategies. The algorithms HHpred [36], M4T [37], RaptorX [38] and SwissModel [39] were used for comparative modeling, while Rosetta [40], I-Tasser [41] and Phyre2 [42] algorithms were used for both *ab initio* and comparative modeling. The generated models were structurally aligned to the BDNF crystallographic structure (PDB ID: 1BND) using the TM-align server. The most accurate structure was selected based on the root-mean-square-deviation (RMSD) < 2.0 Å and TM-score values closer to 1 [20].

### Structure validation

The selected structure had its quality validated by PROCHECK [43], ProSa [44], Verify-3D [45], RAMPAGE [46], and QMEAN [47] servers. We predicted the secondary structure of BDNF using CONCORD [48]. To further validate the *in silico* modeled structure of BDNF, we compared its secondary structure with the 1BND experimental fragment and with the CONCORD prediction [48].

### Phylogenetic analysis

The ConSurf [49] server was used to calculate the evolutionary conservation degree of each amino acid of BDNF. The following parameters were selected for phylogenetic analysis: homologous search algorithm: CSI-BLAST; number of iterations: 3; E-value cut-off: 0.0001; protein database: UNIREF-90; number of reference sequences selected: 150; maximum sequence identity: 95%; minimum identity for counterparts: 35%; alignment method: Bayesian; calculation method: MAFFT-L-INS-i; and evolutionary substitution model: best model.

### Molecular dynamics

*Mutator Plugin1*.*3* [50], available in the Visual Molecular Dynamics (VMD) 4.5 software package [51], was used to induce the V66M substitution on the validated model of BDNF.

Molecular dynamics (MD) simulations of wild-type BDNF and its natural variant V66M were performed in triplicates using the GROMACS 2018.2 package [52]. Amber99SB-ILDN [53] was selected as the force field of the simulations. The structures were solvated using TIP3P water in a dodecahedral box with the dimensions of 55.0 x 82.7 x 45.6 Å. The MD simulation system was neutralized by adding 93 Na+ and 96 Cl− ions. The salt concentrations of the system were set to 0.15 mol/l. The system was then minimized in 5000 steps using the steepest descent method.

After system minimization, three other steps were conducted in the MD simulation: NVT (constant number of particles, volume, and temperature), NPT (constant number of particles, pressure, and temperature) and production. The NVT simulation was followed by the NPT simulation at a temperature of 300 K and 1 atmosphere for a duration of 100 ps [20]. In these simulations, all bonds were constrained in the protein to promote position restraint of this group. The position-restrained NVT and NPT prompted water relaxation around the protein and reduced system entropy. Parrinello-Rahman was selected as the barostat of the MD, whereas v-rescale was selected as the thermostat. The barostat and thermostat relaxation lasted 100 ps each.

The production simulation was performed at 300 K for 200 ns for the wild-type BDNF and its V66M variant. The LINCS (linear constraint solver) algorithm was applied to constrain covalent bonds [54]. The electrostatic interactions were processed using the particle mesh Ewald (PME) method [55]. A time step of 0.002 ps was selected for the simulations, and no constant forces were applied. The MD trajectories were recorded every 10 ps [20].

### Trajectory analysis

Root-mean-square deviation (RMSD), root-mean-square fluctuation (RMSF), B-factor, radius of gyration (Rg), solvent accessible surface area (SASA), and secondary structure were calculated separately for each triplicate trajectory, taking the initial structure of the production dynamics as the reference, to investigate biochemical and structural changes of the native and mutant structures. The following GROMACS distribution programs were applied to perform these analyses: *gmx rms*, *gmx rmsf*, *gmx gyrate*, *gmx sasa*, *and gmx do_dssp*. The means and their respective confidence interval (0.95) were calculated for each triplicate in the RMSD, Rg and SASA analyses using the ggplot2 package in R software [56]. PCA analysis was carried out on all systems using the Bio3D library implemented in R [57]. The full three independent MD simulations were concatenated, and trajectory analyses were conducted for native and mutant structures systems. Rotational and translational motions were removed before calculation of the covariance matrix by least-squares superposition to the corresponding average structures. The 3N×3N covariance matrices of Cα atomic positions (Cartesian coordinates) were then calculated for each state.

VMD software was used to calculate distances between atoms and determine hydrogen bond formation using a geometrical criterion. A hit was computed when the distance between two polar heavy atoms, with at least one hydrogen atom attached, was less than 3.5 Å using a D-Ĥ-A angle cutoff of 30°.

## Results

### Functional and stability prediction analysis

PhD-SNP, PMut, PolyPhen-2, SIFT, SNAP, SNPS&GO, and Varmod were used to predict putative deleterious or neutral effects of the V66M mutation on protein function [24]. The variant V66M was predicted as deleterious by SIFT and PolyPhen-2, whereas it was predicted as neutral by PhD-SNP, PMut, SNAP, SNPS&GO, and Varmod.

SNPeffect 4.0 is a four-algorithm method used for phenotyping human mutations [22]. The algorithms WALTZ, TANGO, and LIMBO were used to predict the effects of the V66M mutation on aggregation tendency (TANGO), amyloid propensity (WALTZ) and chaperone binding tendency (LIMBO). The results indicated that the V66M mutation does not alter these characteristics.

FoldX, the fourth SNPeffect 4.0 algorithm, and I-Mutant2.0 algorithms were used to predict the effects of V66M mutation on protein stability. According to FoldX, the V66M mutation does not change protein stability (ddG = -0.04 kcal/mol), whereas for I-Mutant, the V66M mutation decreases protein stability (ddG = -0.49 kcal/mol).

### Structural modeling

The 1BND structure from the Protein DataBank was selected as template for the structural alignment in TM-align because it has the same sequence coverage and better resolution [27] (2.30 Å) than the only other BDNF structural fragment (1B8M, 2.75 Å) [17].

The generated models using HHpred [36], M4T [37], RaptorX [38], SwissModel [39], Rosetta [40], I-Tasser [41] and Phyre2 [42] were structurally aligned to the 1BND crystallographic fragment of BDNF on the TM-align server. The alignment provides two distinct values: RMSD and TM-score, which are parameters used to evaluate structural similarity [58]. Accurate models present TM-scores approaching 1 and RMSD < 2.0 Å [20]. Table 1 shows the RMSD and TM-Score values of the structural alignment.

**Table 1:** Structural alignment of the generated models of BDNF and the 1BND fragment.

Modeling Algorithms	RMSD (Å)	TM-Score
HHPred	0.53	0.98489
I-Tasser	1.40	0.89691
M4T	1.21	0.93869
Phyre2	1.65	0.90354
RaptorX	1.21	0.93869
Rosetta	1.04	0.94492
SwissModel	1.05	0.95347

The algorithms generated accurate models according to the RMSD and TM-scores presented in Table 1. HHPred and SwissModel structures presented the best scores, but these structures were not considered for further analyses because these algorithms generated incomplete models of BDNF.

**Fig 1:**
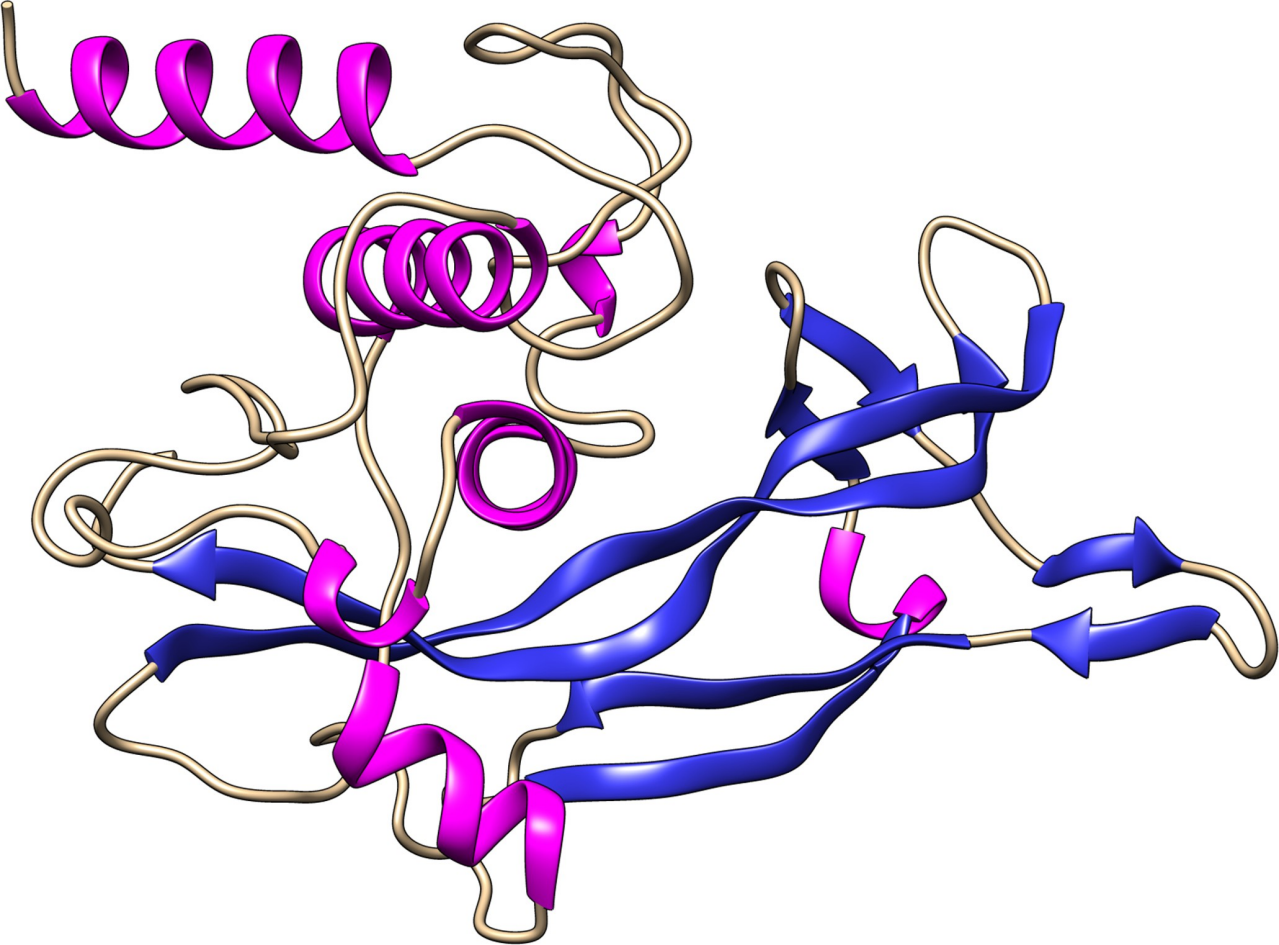
*In silico* modeled structure of BDNF by Rosetta. Secondary structure representation of the *in silico* modeled structure of BDNF generated by Rosetta. The α-helices are represented by pink ribbons, the β-strands are represented by blue arrows, and the coiled regions are represented in beige.

**Fig 2:**
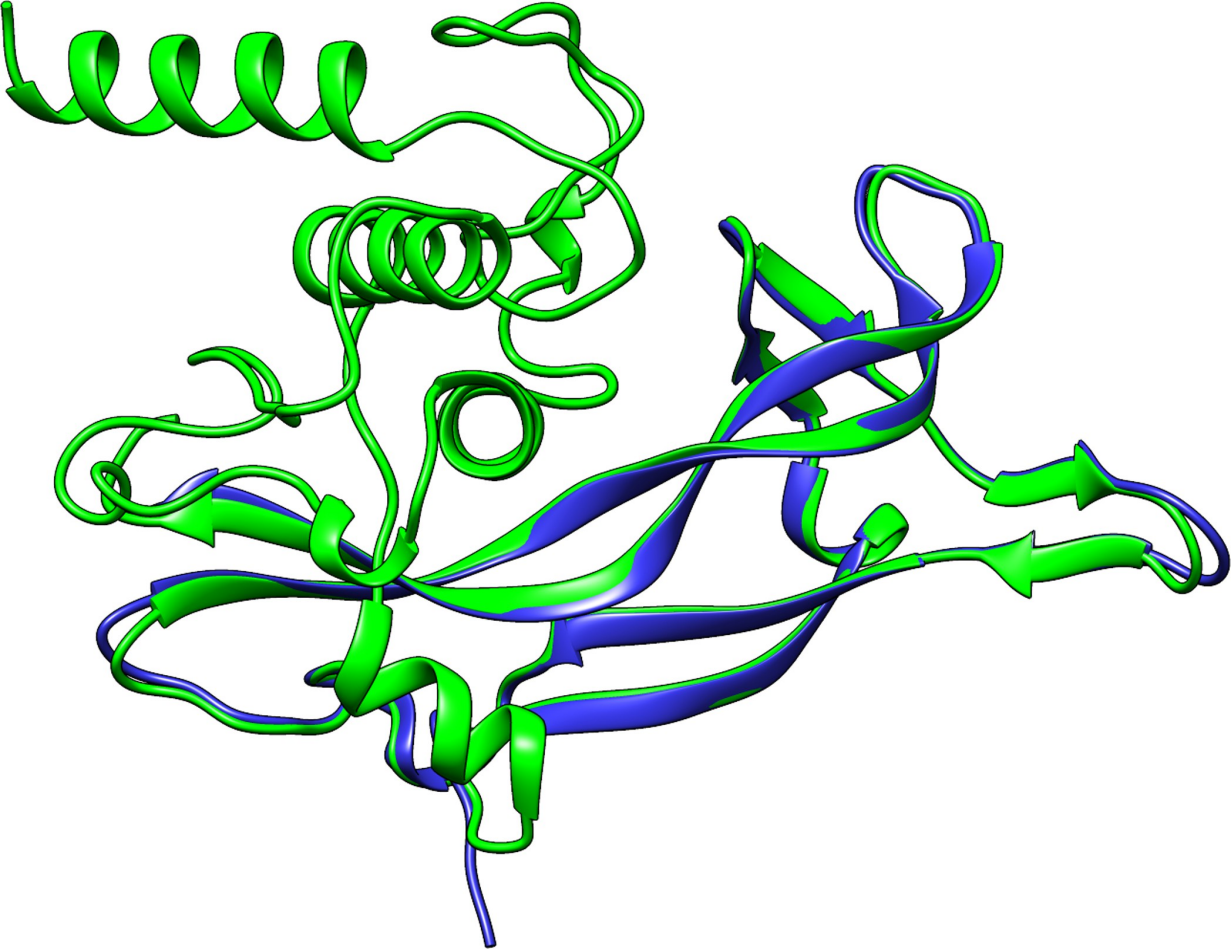
Structural alignment between the theoretical model of BDNF, generated by the Rosetta algorithm, and the experimental fragment of BDNF (PDB ID: 1BND). Cartoon representation of BDNF modeled structure created by Rosetta (green) and the experimental fragment 1BND (blue). The α-helices are represented by ribbons, while the β-strands are represented by arrows.

Amongst the complete generated BDNF structural models, the one provided by Rosetta (Fig 1) presented similar structure to its template and RMSD of 1.04 Å and a TM-score of 0.94492 (Fig 2). Therefore, the Rosetta model was considered the most accurate and selected for structural validation.

### Structure validation

The Rosetta structure had its quality validated by PROCHECK [43], ProSa [44], Verify-3D [45], RAMPAGE [46], and QMEAN [47] servers.

**Fig 3:**
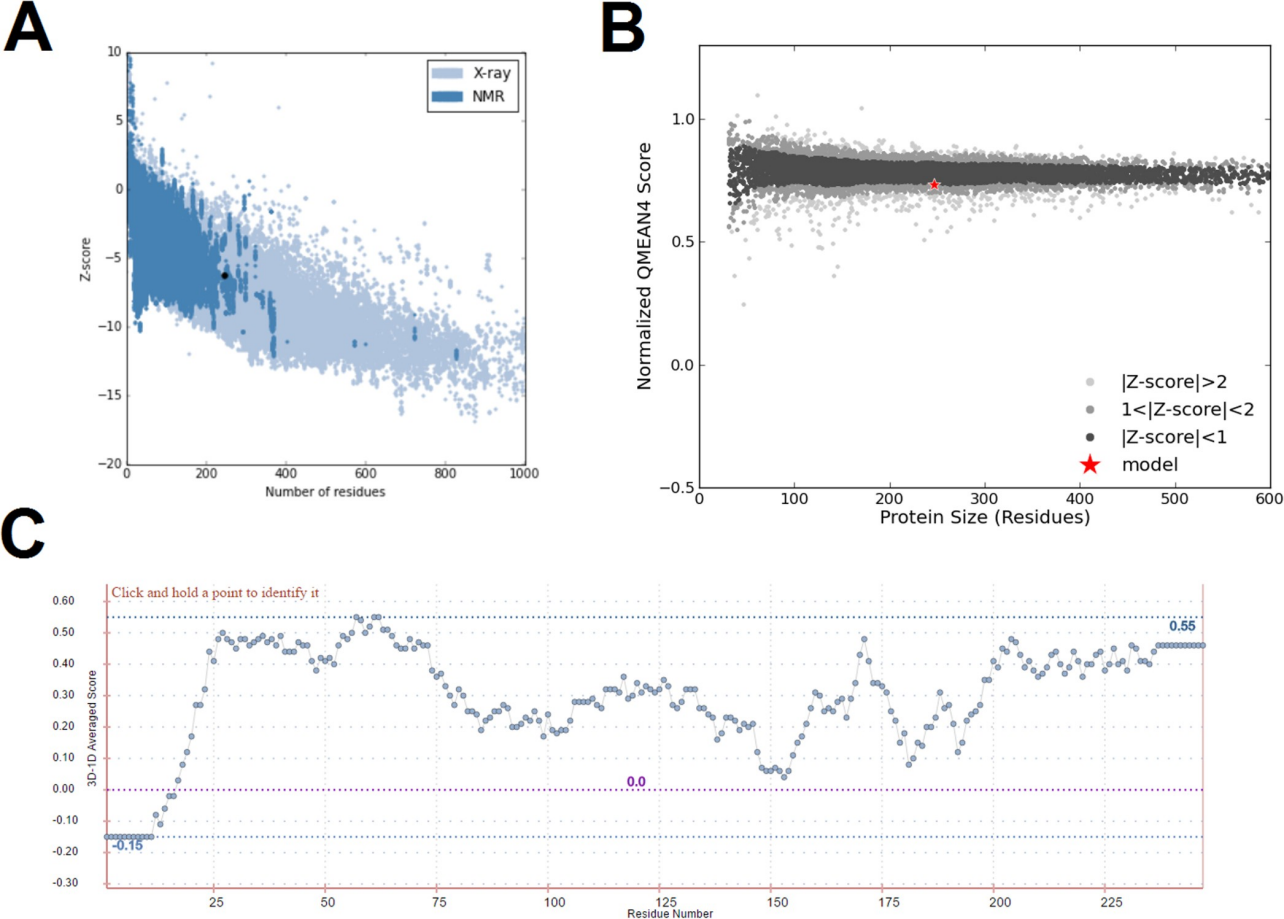
Validation of the *in silico* predicted model of BDNF by ProSa, QMEAN, and Verify3D. (A) Structure validation by ProSa, which shows the Z-score of the predicted model of BDNF (black dot), when compared to a non-redundant set of crystallographic structures (light blue dots) and NMR structures (dark blue dots). The *in silico* structure of BDNF presents an overall quality score comparable to that of NMR structures. (B) Structure validation by QMEAN, which shows the QMEAN-score of the predicted model of BDNF (red star), when compared to a non-redundant set of high-resolution experimental structures (gray and black dots). (C) Structure validation by Verify3D, which shows the 3D-1D score for each atom of the predicted model of BDNF. The graphic shows that 80.57% of the residues of the *in silico* structure of BDNF presented a compatibility score of 0.2 or higher, which indicates that the structure is a high-quality model according to Verify3D.

ProSa evaluates the quality of the submitted model by calculating its potential energy and comparing the obtained score with those of the experimental structures deposited in PDB [44]. According to ProSa, the *in silico* structure of BDNF presented a Z-score of -6.24, which is comparable to that of NMR structures of the same size (Fig 3A).

QMEAN estimates the quality of the submitted model based on its physicochemical properties and then generates a value referring to the overall quality of the structure and set it against the calculated QMEAN-scores of 9766 high-resolution experimental structures [47]. According to QMEAN, the *in silico* structure of BDNF presented a QMEAN-score of -1.13, which is comparable to that of high-resolution experimental structures (Fig 3B).

Verify3D assesses the local quality of the submitted model based on its structure-sequence compatibility to generate a compatibility value for each residue of the protein. High-quality structures are expected to present more than 80% of their residues with a 3D-1D score equal to or higher than 0.2 [45]. The *in silico* structure of BDNF presents 3D-1D scores of 0.2 or higher (Fig 2C) in 80.57% of its residues, thus it is considered a high-quality model.

**Fig 4:**
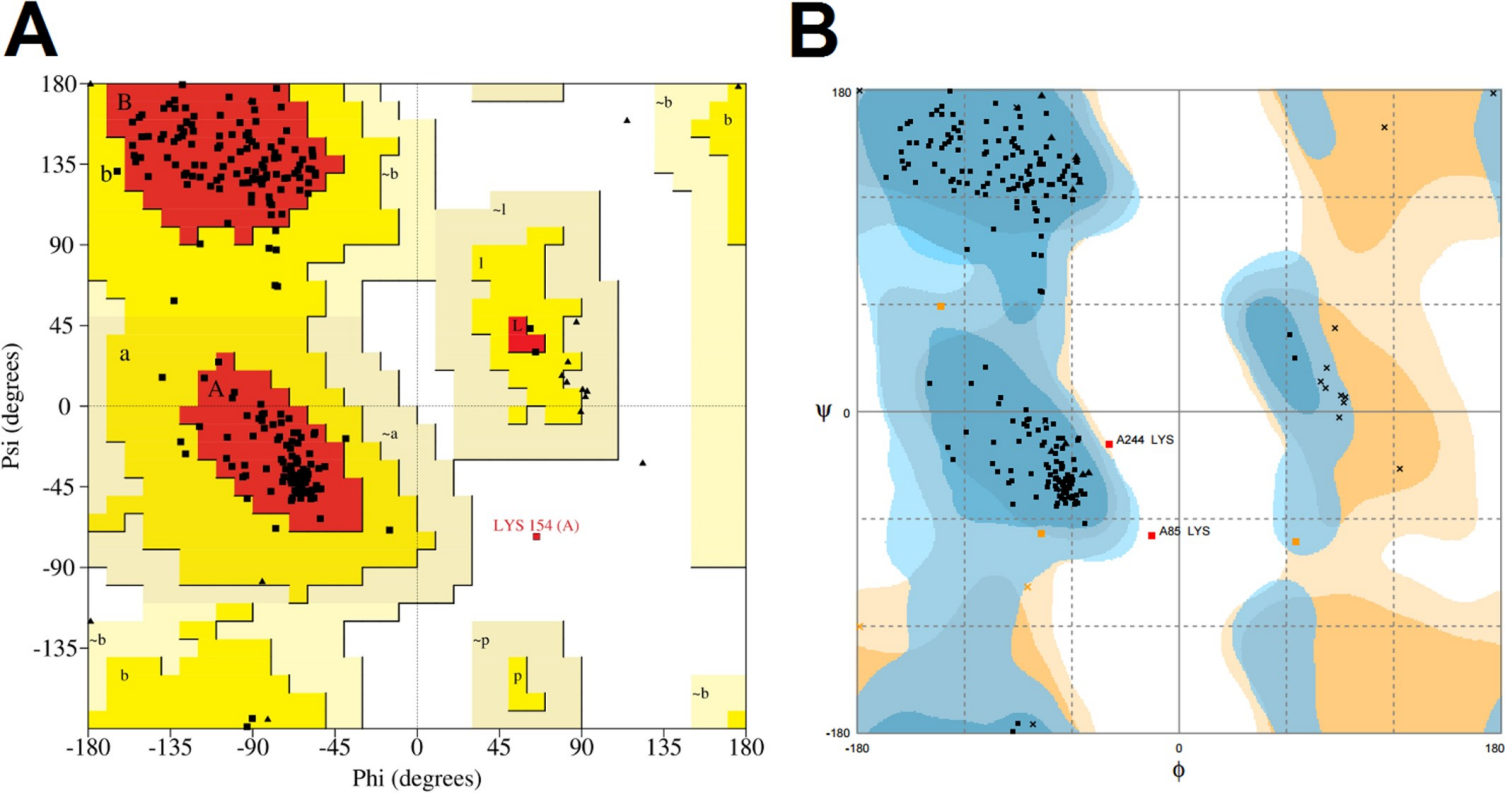
Validation of the *in silico* predicted model of BDNF by PROCHECK and RAMPAGE. (A) PROCHECK’s Ramachandran plot shows that the *in silico* structure of BDNF. Most residues (90.8%) are located in the most favored regions, while 8.8% are in additional allowed regions, and only 0.5% in disallowed regions, configuring a high-quality model. (B) RAMPAGE’s Ramachandran plot of the *in silico* structure of BDNF. This structure is also considered as a high-quality model because it shows 97.1% of its residues on favored regions, 2.0% on allowed regions, and 0.8% on not allowed regions.

PROCHECK and RAMPAGE evaluate the stereochemical quality of the submitted models based on its phi/psi angle arrangement and then generates Ramachandran plots in which the protein residues are placed on favored, allowed and not allowed regions. For PROCHECK and RAMPAGE, high-quality models are expected to have more than 90% of their residues in the most favored regions of PROCHECK’s Ramachandran plot and approximately 98% of their residues in the favored regions of RAMPAGE’s Ramachandran plot [43,46]. The predicted model of BDNF shows 90.8% of its residues in the most favored regions of PROCHECK’s Ramachandran plot and 97.1 of its residues in the favored region of the RAMPAGE’s Ramachandran plot. Thus, it is considered a high-quality model (Fig 4).

**Fig 5:**
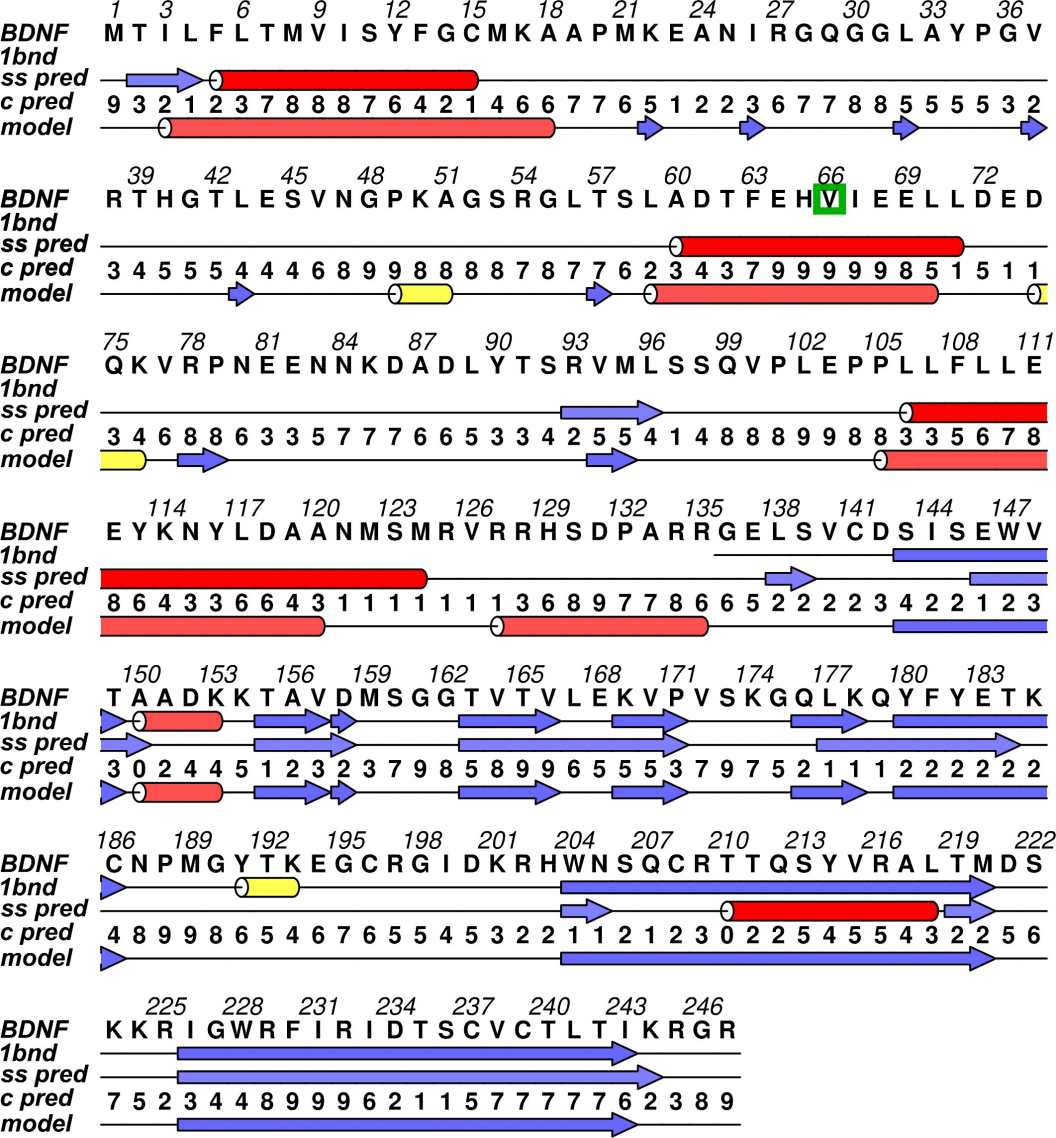
Secondary structure comparison between the theoretical model of BDNF, the CONCORD prediction, and the 1BND experimental fragment. The amino acid sequence of BDNF wild type is shown in the first line of the alignment, followed by the secondary structure of the 1BND fragment, the consensus secondary structure prediction by CONCORD (ss pred), the CONCORD confidence score (c pred), and the secondary structure of the theoretical model of BDNF. Red cylinders represent the α-helices, yellow cylinders represent the 3–10 helices, and blue arrows represent the β-strands.

Moreover, the secondary structure of human BDNF was predicted by CONCORD, a consensus method that is based on seven secondary structure prediction methods: SSpro, DSC, PROF, PROFphd, PSIPRED, Predator, and GorIV [48]. To further validate the *in silico* modeled structure of BDNF, we compared its secondary structure with the 1BND experimental fragment and with the CONCORD results. This comparison is shown in Fig 5, and indicates that the theoretical model of BDNF also has a high accuracy regarding its secondary structure. The ALINE software [59] was used to generate the secondary structure comparison scheme.

With the quality assessment and the confirmation of the secondary structure prediction, the Rosetta’s model was considered a high-quality model. Thus, it was selected for the subsequent analyses.

### Phylogenetic analysis

ConSurf is a bioinformatics tool that applies statistical inference methods, machine learning and multiple sequence alignment to estimate the evolutionary conservation of amino acids in a protein. ConSurf then attributes a conservation score to each residue based on the phylogenetic relationship between the protein and its homologous sequences. The conservation scores are associated with a coloring scheme and projected on the protein’s surface [49].

**Fig 6:**
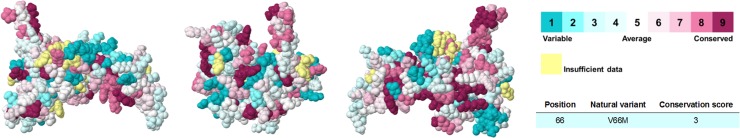
Evolutionary conservation analysis of BDNF. The BDNF conservation profile is shown in three different angles. The amino acid residues are represented as space-filling models and colored according to their conservation scores. The color coding bar shows the ConSurf conservation scores. The amino acid residues colored in yellow did not receive conservation scores due to insufficient data. According to ConSurf, position 66 of BDNF is variable and buried. Highly conserved positions are colored maroon, intermediately conserved residues are colored white and variable positions are colored turquoise.

The validated model of BDNF was submitted to ConSurf, which calculated the evolutionary conservation score of each amino acid of BDNF (Fig 6).The position 66 of BDNF was classified as variable and buried.

### Molecular dynamics

MD simulations compute the time-dependent motion of a molecular system based on concepts of physics, chemistry and mathematics [60,61]. Thus, the MD approach can be used to reproduce the real behavior of a protein in its environment and to calculate its trajectory over time [20]. These simulations provide detailed information on changes in protein conformation and fluctuations that can be used to assess biochemical and structural parameters, such as flexibility and stability [60].

**Fig 7:**
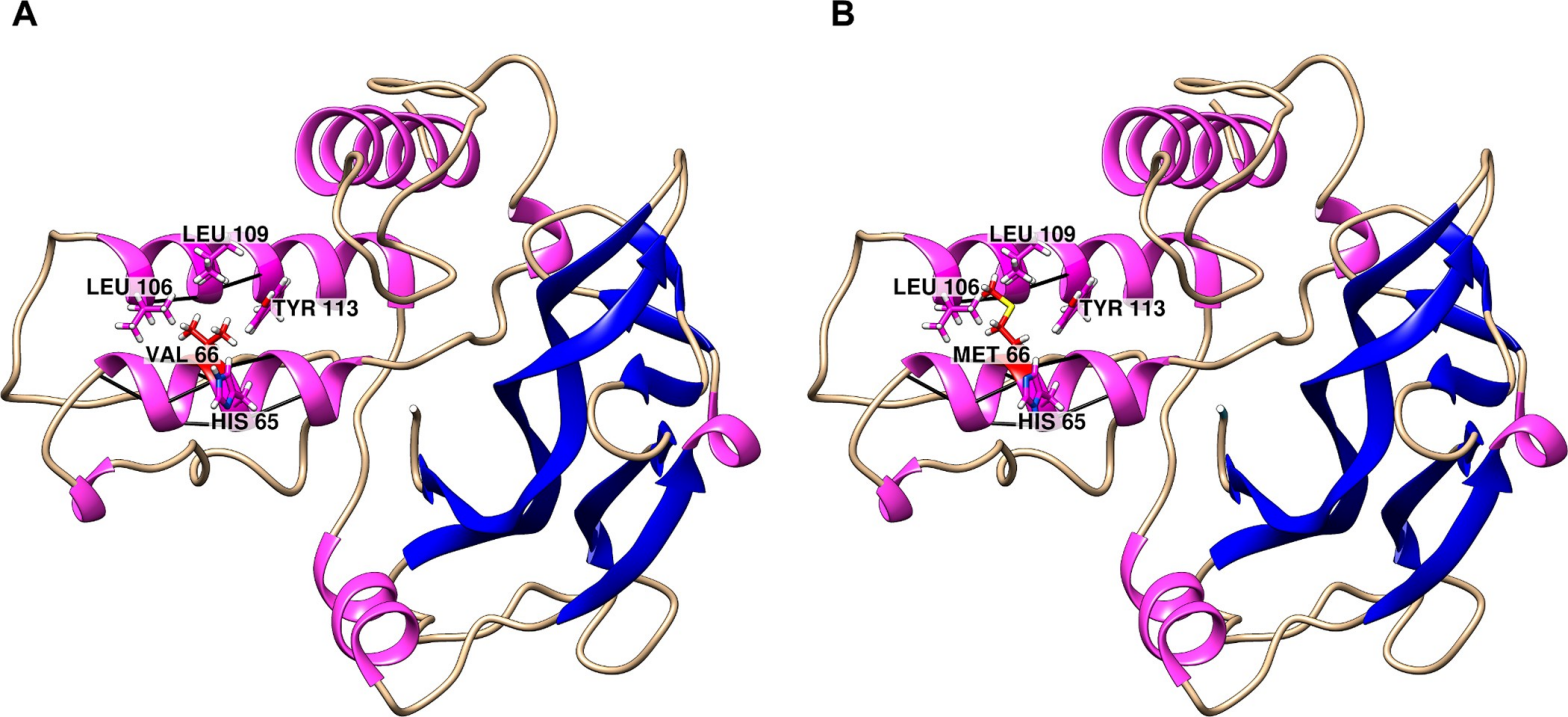
Complete theoretical models of wild-type BDNF and its variant V66M. Secondary structure representation of the *in silico* modeled structures of wild-type BDNF and its variant V66M. The α-helices are represented by pink ribbons, the β-strands are represented by blue arrows, and the coiled regions are represented in beige. Residues within 5 Å of val66 and met66 are shown in the sticks. Black lines represent hydrogen bonds. (A) Wild-type BDNF. (B) Variant V66M.

The impact of mutations on the structure and function of a protein can be examined using MD simulations [62]. The complete theoretical models of wild-type BDNF and its V66M variant (Fig 7) were submitted to MD simulations on GROMACS. The simulations were performed in triplicates and the obtained results were analyzed comparatively in order to understand the effects of the V66M mutation on BDNF structure and function. The means and their respective confidence intervals (0.95) were calculated for each triplicate in the RMSD, Rg and SASA.

RMSD and RMSF are common measures of structural fluctuations [61,63]. For purposes of MD, RMSD is defined as the root mean square deviation of the atoms of a structure at a given instant of the simulation relative to its initial position in the MD trajectory [63]. RMSD is therefore useful for analyzing the time-dependent motion of a given structure and for determining its spatial convergence throughout the simulation. A plateau of RMSD values indicates that the structure fluctuates around a stable average conformation. Thus it makes sense to evaluate its local fluctuations [61,63].

**Fig 8:**
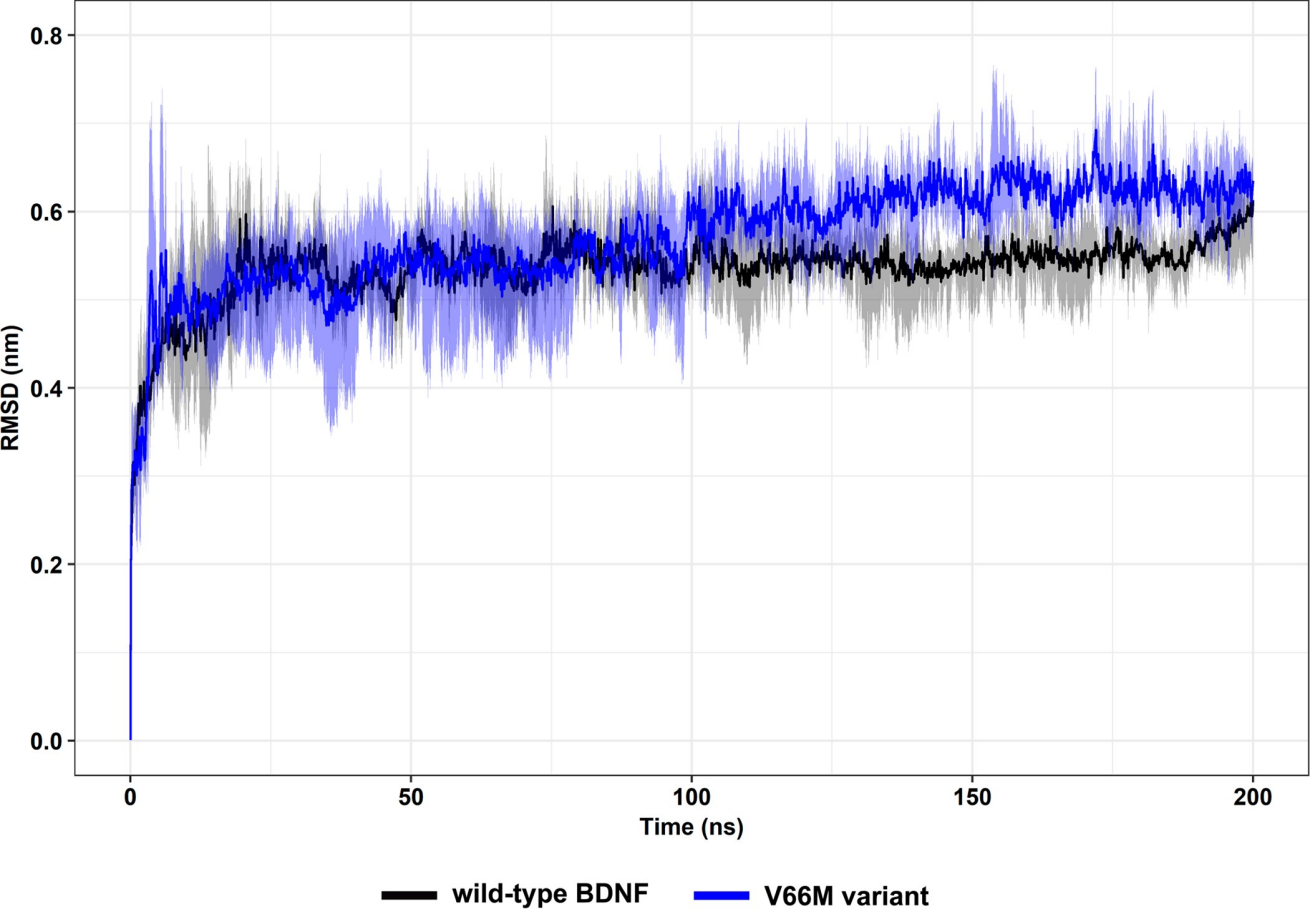
Backbone RMSD of wild-type BDNF and its V66M variant. RMSD of the backbone atoms of wild-type BDNF (black) and its V66M variant (blue) structures at 300 K are shown as a function of time. Mean (solid lines) and confidence interval (smooth lines) of the replicates are displayed.

We analyzed the time evolution of backbone RMSD values to monitor the overall stability of each model during 200 ns MD in explicit solvent. Wild-type and V66M systems reached equilibrium in approximately 50 ns and 100 ns, respectively, and presented a steady behavior throughout the triplicates (Fig 8). Furthermore, the RMSD values of the V66M variant at the plateau (0.62 ± 0.04 nm) are similar to those of the wild-type structure (0.55 ± 0.04 nm).

The RMSD values distribution only provides information about the overall structure [64]. Thus, RMSF analysis, which assesses the deviations of an atom or group of atoms from its average position in a given structure [63,64], is often applied to obtain local information. This analysis is therefore useful to describe local flexibility, thermal stability, and heterogeneity of macromolecules [64] based on residue displacement during the MD simulation [20].

**Fig 9:**
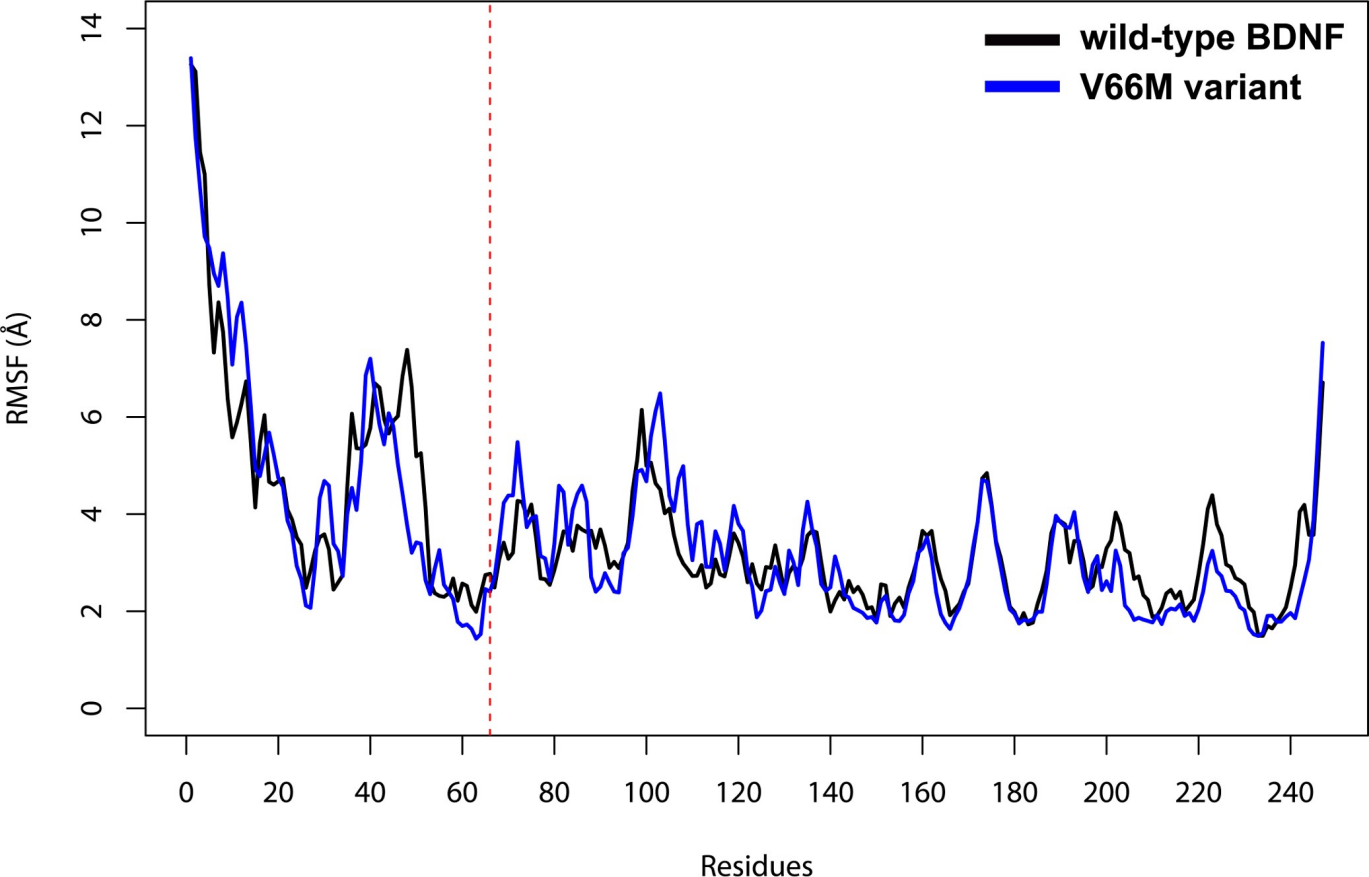
RMSF of wild-type BDNF and its V66M variant. RMSF of each residue of wild-type BDNF (black) and its V66M variant (blue) at 300 K are shown as a line plot. The dashed red line shows residue 66.

The comparison of the fluctuations between wild-type and V66M variant structures evidenced that the presence of the mutation resulted in no significant local flexibility alterations (Fig 9).

Structural flexibility can also be assessed during an MD simulation by analyzing the B-factor, a measure of atomic displacement due to thermal vibrations [65]. B-factor, as well as RMSF, reflects the fluctuation of an atom or a set of atoms around their average position [64,66]. B-factor is therefore used in most methods of predicting protein flexibility [65] and applied to predict protein flexibility [64].

**Fig 10:**
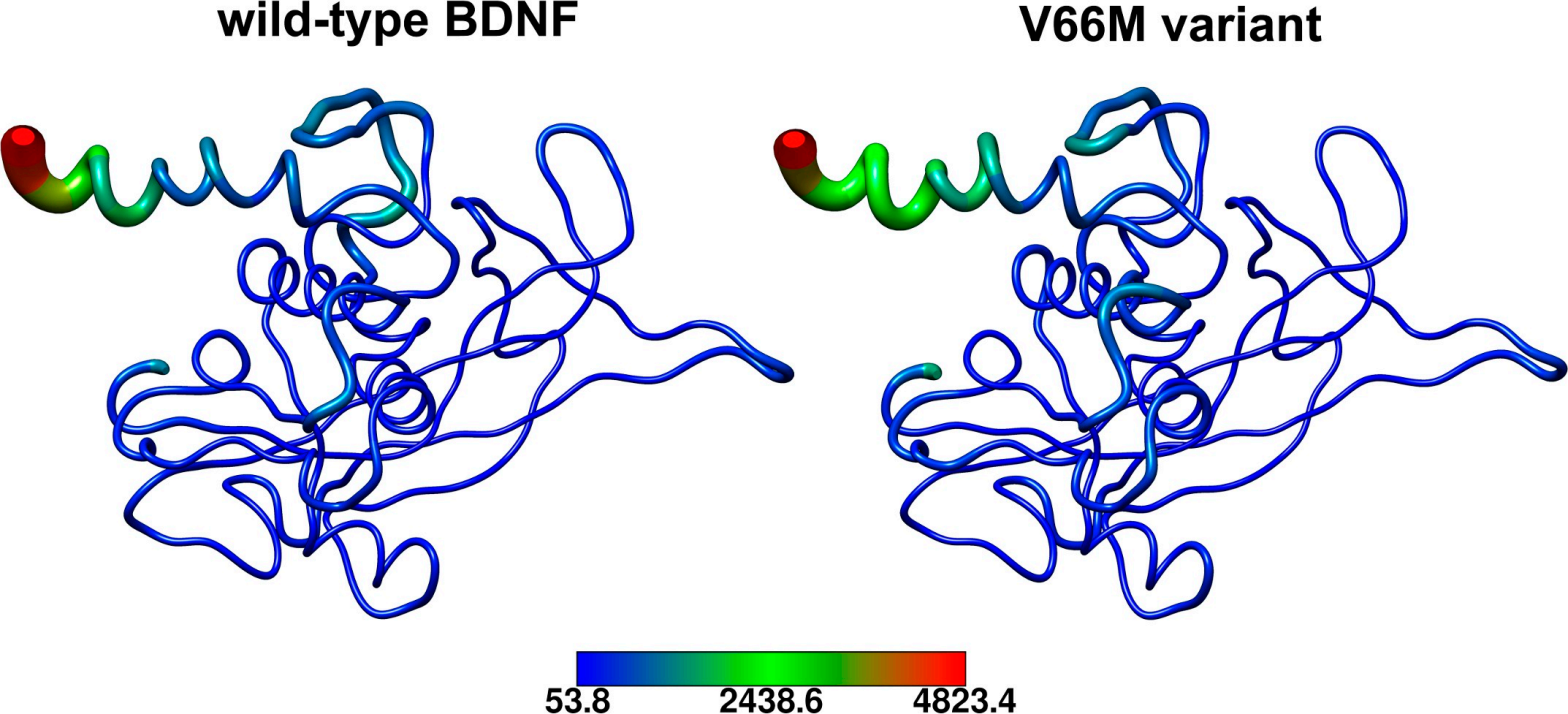
B-factor representation of wild-type BDNF and its V66M variant. The B-factor values of each residue of wild-type BDNF and its V66M variant expressed in a coloring-thickness scheme. Higher B-factor values are represented as red and bulky structures, while lower B-factor values are represented as dark blue and thin structures.

An overview of structural mobility indicated that the flexibility of the wild-type and V66M variant of BDNF are similar (Fig 10). B-factor analysis indicated that the V66M mutation does not affect BDNF local flexibility.

The biological function of proteins is associated with their molecular motions. Therefore, a thorough understanding of protein function requires the investigation of its static structures, as well as its dynamics and molecular motions [67].

Protein dynamical mechanical properties are usually best characterized by analyzing its phase space behavior [62]. PCA, also known as essential dynamics (ED), is a statistical technique that extracts the essential motions of a protein from a set of sampled conformations using covariance or correlation matrices. Thus, PCA reduces the number of dimensions needed to describe protein dynamics [68]. The protein fluctuations are confined within the eigenvectors of the covariance matrix, which are also called principal components. Projections of the protein trajectories on the eigenvectors thus describe their essential motions in phase space [62].

**Fig 11:**
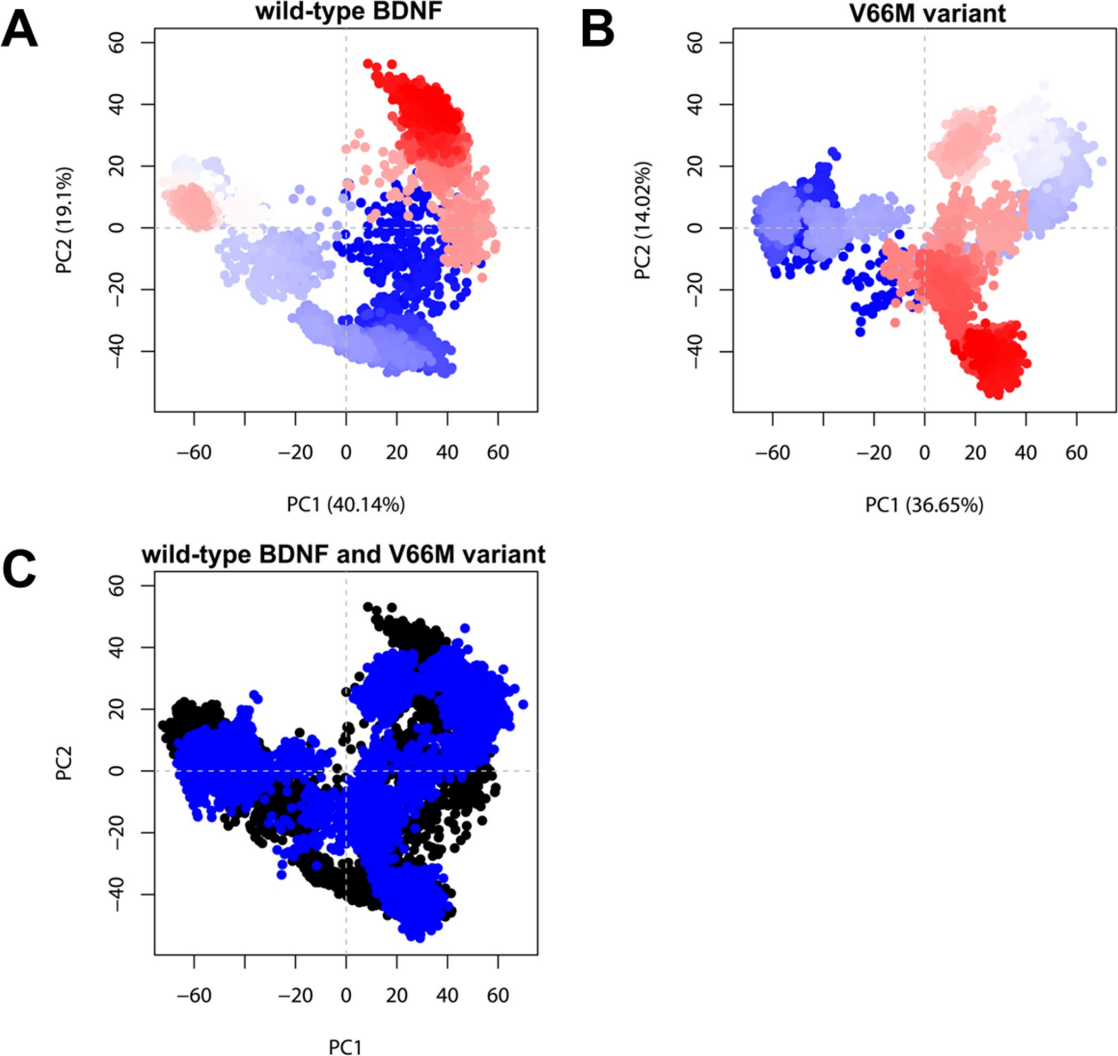
Principal component analysis (PCA) of wild-type BDNF and its variant V66M. The plots are showing the first two principal components (PC1 and PC2) extracted from the essential dynamics. The trajectory frames for wild-type BDNF and its variant V66M were colored from blue to red according to time evolution. (A) PCA plot of wild-type BDNF. The first two PCs account for 59.24% of the total structural variance. (B) PCA plot of variant V66M. The first two PCs account for 50.67% of the total structural variance. (C) PCA comparison between wild-type BDNF (black) and its variant V66M (blue).

**Fig 12:**
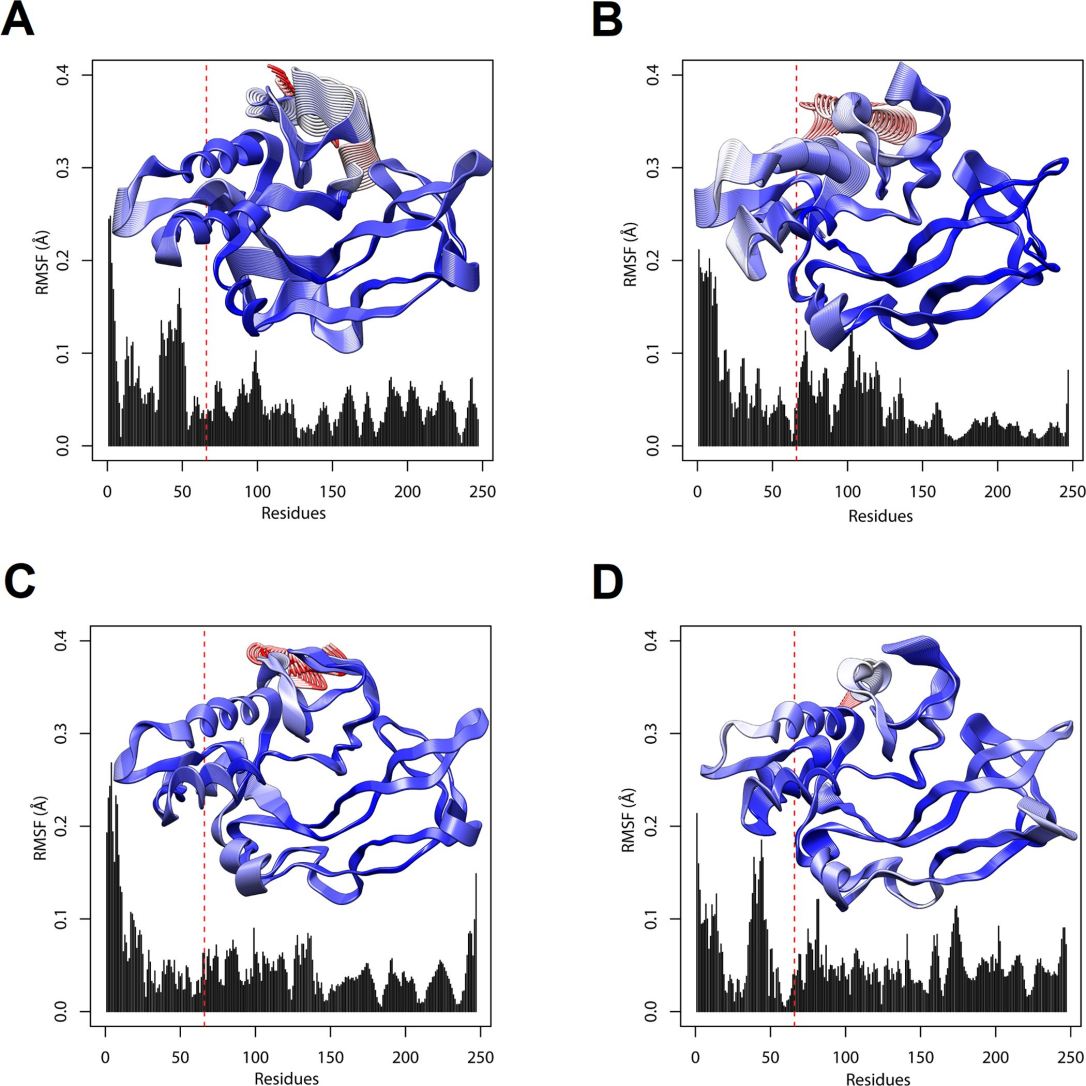
Visualization of the first two principal components on the structure of wild-type BDNF and its variant V66M. The RMSF contribution of each protein residue to the first two principal components (PC1 and PC2) is displayed within their respective plot and projected on the corresponding structural location. RMSF magnitude is represented by a coloring-thickness scheme, which scales from thin and blue (low fluctuations) to thick and red (large fluctuations). The dashed red lines show residue 66. (A) RMSF contribution of wild-type to PC1. (B) RMSF contribution of variant V66M to PC1. (C) RMSF contribution of wild type to PC2. (D) RMSF contribution of variant V66M to PC2.

The ED method can be used to study the influence of residue mutations on molecular motions [67]. Therefore, we performed PCA on the snapshots obtained from the full MD trajectories to identify statistically relevant motions (essential motions) [69] of wild-type BDNF and its V66M variant in solution. The first two components (named PC1 and PC2) captured the dominant motions, accounting for 59.24% and 50.67% of the overall variance in wild-type BDNF and V66M variant, respectively. We compared the projections of the trajectories onto the subspace spanned by the first two principal components (Fig 11), as well as analyzed the RMSF contribution of each protein residue to the first two principal components (Fig 12). Overall, the variant V66M exhibited altered essential mobility when compared to the wild-type BDNF, especially at its N-terminus, suggesting that the V66M mutation could affect the BDNF essential motions.

**Fig 13:**
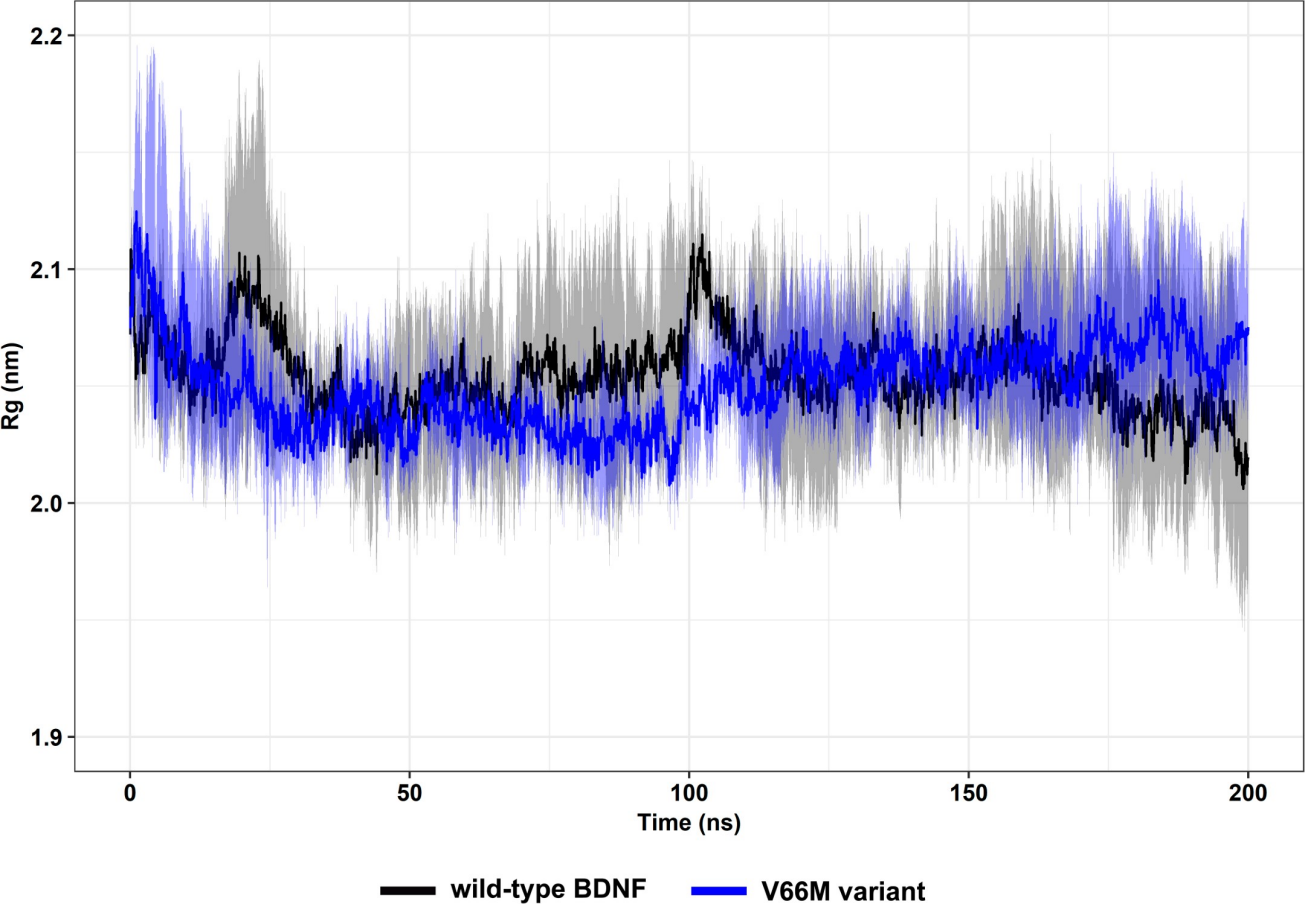
Rg of wild-type BDNF and its V66M variant. Rg of the Cα atoms of wild-type BDNF (black) and its V66M variant (blue) at 300 K are shown as a function of time. Mean (solid lines) and confidence interval (smooth lines) of the replicates are displayed.

Rg is the mass-weighted root mean square distance of a group of atoms from their common center of mass [62] and is used to describe the dimensions of proteins and other biopolymers [64]. Rg analysis, therefore, generates comprehensive information on protein compactness during an MD [70]. The overall dimensions of the wild-type and V66M variant systems presented an average Rg of 2.05 ± 0.04 nm and 2.05 ± 0.03 nm, respectively (Fig 13), suggesting that the V66M mutation does not affect the BDNF compactness.

**Fig 14:**
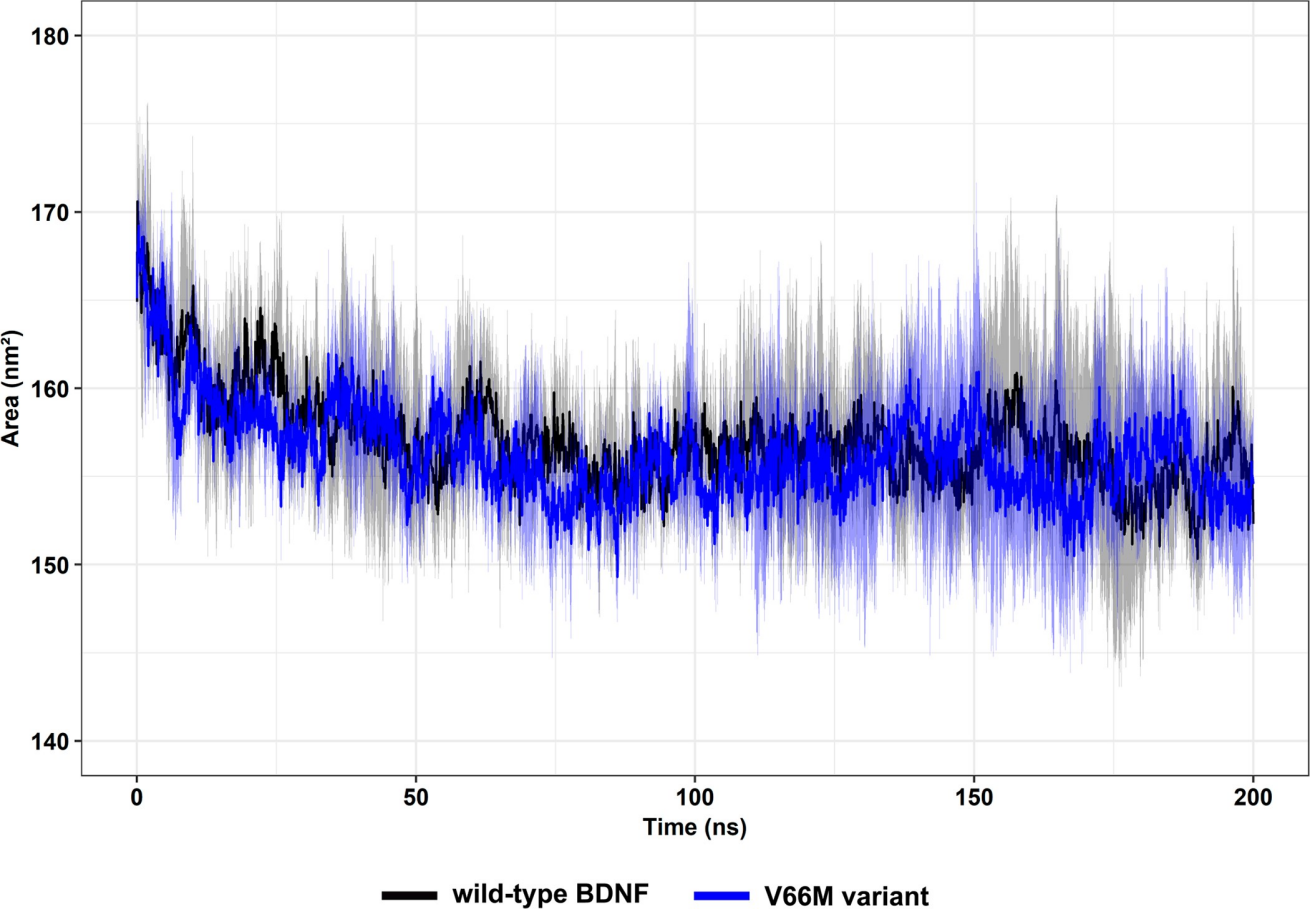
SASA of wild-type BDNF and its V66M variant. SASA of wild-type BDNF (black) and its V66M variant (blue) are shown as a function of time. Mean (solid lines) and confidence interval (smooth lines) of the replicates are displayed.

Solvent accessible surface area (SASA) is defined as the exposed surface of a protein that can be accessed by a solvent [71,72]. Thus, SASA analysis is useful to understand the protein’s ability to interact with solvents [72] and other molecules. The accessible surface area of the wild-type and V66M variant systems are similar, presenting an average SASA of 157.08 ± 4.82 nm^2^ and 156.25 ± 4.31 nm^2^, respectively (Fig 14). This analysis suggests that no significant variations in SASA were observed during the simulations.

**Fig 15:**
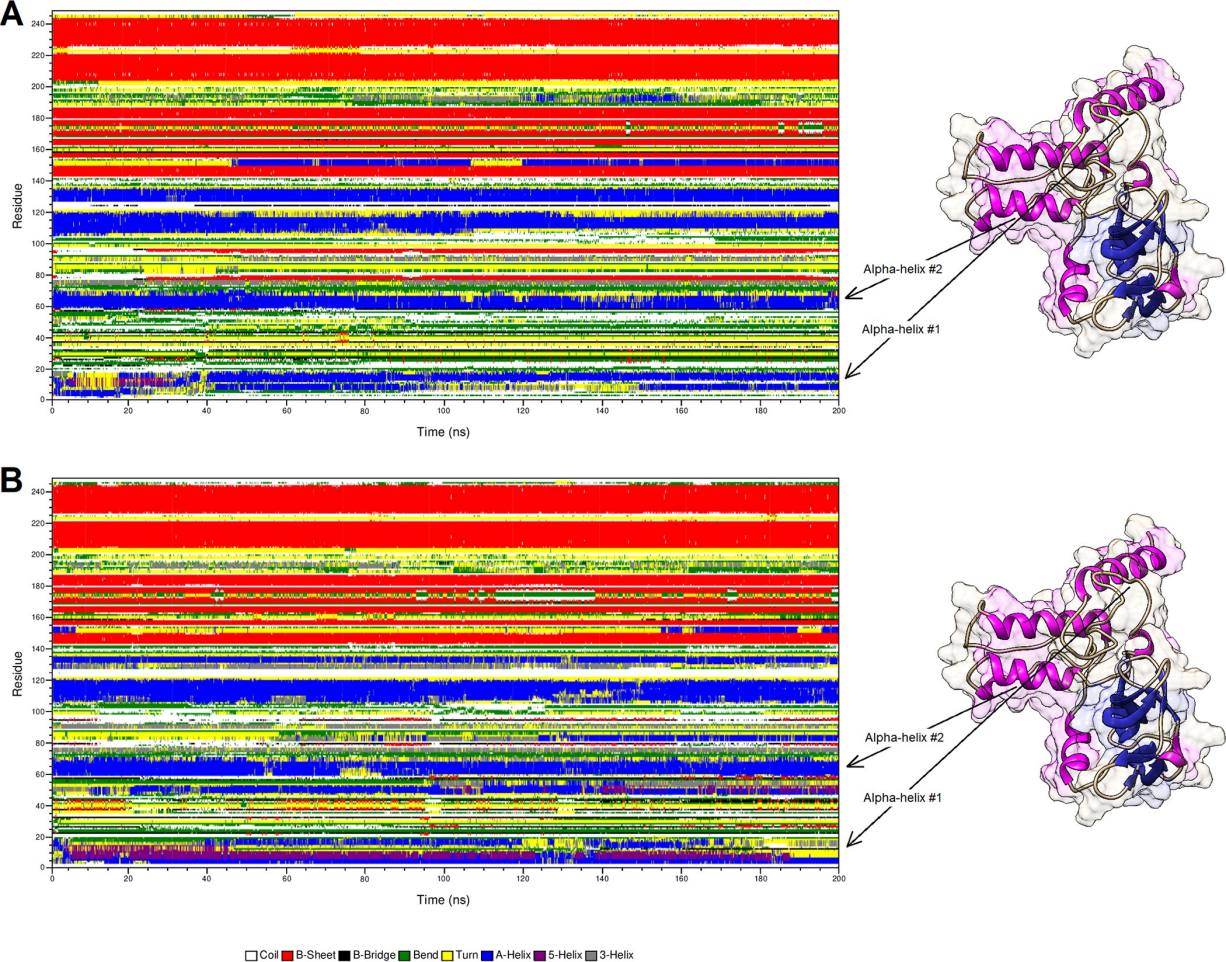
Secondary structure analysis of BDNF wild-type and its V66M variant. Time evolution of secondary structural elements during the MD simulations of wild-type BDNF (A) and its V66M variant (B). Coils are represented in white, β-sheets are represented in red, β-bridges are represented in black, bends are represented in green, turns are represented in yellow, α-helices are represented in blue, 5-helices are represented in purple and 3-helices are represented in gray.

The function of most proteins is dependent on the interaction and recognition of other molecules. In addition, much of the binding selectivity comes from the hydrogen-bonding formation between a given protein and its molecular partner [73]. The secondary structure is also a well-known factor for protein interactions [74,75], since this structural pattern may both favor or disfavor interactions, such as hydrogen-bonding [73]. We then analyzed the pattern of secondary structure (Fig 15), as well as the hydrogen-bonding formation of the residue 66 (Table 2) during the MD simulations for BDNF wild-type and its V66M variant [14].

**Table 2:** Hydrogen bond occupancy of the residue 66 during the MD simulations of BDNF wild-type and its V66M variant.

Wild-type BDNF			V66M BDNF		
Donor	Acceptor	Occupancy (%)	Donor	Acceptor	Occupancy (%)
VAL66-Main-N	THR62-Main-O	92.12	MET66-Main-N	THR62-Main-O	0
VAL66-Side-CG2	THR62-Main-O	16.29	MET66-Side-CG	THR62-Main-O	7.29
LEU70-Main-N	VAL66-Main-O	24.25	LEU70-Main-N	MET66-Main-O	10.16
GLU69-Main-N	VAL66-Main-O	17.75	GLU69-Main-N	MET66-Main-O	10.59
VAL66-Main-N	PHE63-Main-O	17.95	MET66-Main-N	PHE63-Main-O	32.96

Atom pairs with less than 5% occupancy are not shown in the table.

The secondary structure analysis pointed to alterations in the pattern of secondary structure of BDNF wild-type when compared to its V66M variant, especially at the first alpha-helix of the protein, comprised between the residues 3 and 18. The second alpha-helix of BDNF, which contains the position 66 and adjacent residues, presented little alterations when compared to the wild type, particularly between 68 and 78 ns. It suggests that the V66M mutation could affect the BDNF secondary structure especially at its first alpha-helix (BDNF pre-domain), but also may affect the BDNF pro-domain (position 66 and adjacent residues) [76].

The hydrogen-bonding analysis (Table 2) indicated that the occupancy of the hydrogen bonds formed during the simulations was different for the BDNF wild-type and its V66M variant, especially regarding the hydrogen bonds formed between val66-thr62 and met66-thr62. The occupancy of the hydrogen bond formed between the main chains of val66-thr62 and met66-thr62 is respectively 92.12% and 0%.

## Discussion

BDNF plays a key role in the proliferation, maturation, and maintenance of neuronal function [8,13]. The most frequent missense mutation of BDNF in humans, V66M [13], is believed to impair protein function and distribution [14,15]. This mutation is associated with the development of psychiatric disorders [13].

The pro-domain of BDNF, particularly the position 66 and its adjacent residues, are determinant for the intracellular sorting and activity-dependent secretion of the molecule [14]. This region also acts as a molecular chaperone to assisting the folding [77] of nascent BDNF, in addition to being directly involved in the proteolytic cleavage of pro-BDNF to mature-BDNF [78].

The substitution of a Valine to a Methionine at the position 66 of BDNF occurs at its N-terminal pro-region [77]. This substitution affects the intracellular sorting of BDNF [16] and reduces the amount of the protein targeted to secretory granules [11]. This pathological condition impairs the activity-dependent secretion of BDNF [79], which is the main pathway responsible for the protein release in neurons [80].

In addition to reducing BDNF secretion in neurons [77,80], the V66M mutation also impacts BDNF expression levels [81,82]. Moreover, this mutation is believed to disrupt the folding and dimerization of BDNF [16]. However, the V66M substitution does not affect the efficiency of pro-BDNF processing and, consequently, the formation of mature-BDNF [79].

The pro-domain of BDNF does not appear to participate in the protein dimerization process, since the formation of BDNF dimers depends on a cysteine knot structure linking the mature domains of each protein monomer. Importantly, this connection is maintained even after the cleavage of BDNF pro-domains [83]. However, the V66M mutation is thought to impair BDNF dimerization [16].

The effects of deleterious mutations on proteins can be predicted *in silico* by computational simulations [70]. We used the PhD-SNP [28], PMut[29], PolyPhen-2 [30], SIFT [31], SNAP [32], SNPS&GO [33], VarMod [34], SNPeffect4.0 [22], and I-Mutant2.0 [35] algorithms to predict the V66M effects on the BDNF function and stability.

The V66M variant of BDNF was predicted as deleterious by SIFT and PolyPhen-2, whereas it was predicted as neutral by PhD-SNP, PMut, SNAP, SNPS&GO, and Varmod. This analysis shows little concordance amongst the functional prediction algorithms used in detecting the known deleterious potential of the V66M mutation [13,15], as only two out of the seven algorithms were able to detect it. The available methods were trained in different data sets [84] and present different strategies to predict the putative functional effects of mutations on proteins. Considering that there is no gold-standard method, it is important to use more than one algorithm to determine the deleterious potential of mutations [20,24]. The divergent results reaffirm the need of improving such methods.

The FoldX prediction indicated that the V66M mutation does not affect BDNF stability (ddG = -0.04 kcal/mol), whereas the I-Mutant prediction indicated that the V66M mutation decreases protein stability (ddG = -0.49 kcal/mol). The inconsistency between these results may occur due to the different strategies that the FoldX and I-Mutant algorithms apply to make predictions and classify mutations [35,85]. While FoldX evaluates free energy from the application of an empirical force field that was trained in the molecular interactions of engineered proteins [85], I-Mutant applies Support Vector Machine methods to calculate free energy based on experimentally determined structures [35]. Moreover, FoldX presented three possible classifications for the mutation effects on protein stability: does not affect protein stability (ddG between 0.5 and -0.5 kcal/mol), decreases protein stability (ddG > 0.5) and increases protein stability (ddG < -0.5) [22], whereas for I-Mutant, there are only two possible classifications: decrease protein stability (ddG < 0) or increase protein stability (ddG > 0) [35].

The SNPeffect prediction indicated that the V66M mutation does not affect the aggregation tendency, amyloid propensity and chaperone binding tendency of BDNF.

The three-dimensional protein structure is essential for understanding the mechanisms by which proteins perform their functions [18] and the mechanisms which disease-associated mutations disrupt these functions [19]. In addition to experimental methods for determining protein structures, such as x-ray crystallography and NMR, the structure of a protein can be predicted using theoretical modeling [60]. However, considering that experimental methods of determining protein structures are expensive and time consuming [20] and that massively parallel and low-cost sequencing methods determine protein sequences at a much faster rate [19], theoretical modeling is presented as an efficient [20] and necessary approach to solving the protein structural gap [18] because it enables modeling of three-dimensional structures in a cheaper, faster, and more accurate way [20].

There are two theoretical modeling methods that are worth mentioning: comparative and *ab initio* modeling. *Ab initio* modeling predicts protein structures based on energy functions and sequence information [86], whereas comparative modeling builds three-dimensional protein models using experimentally determined structures as the template [27]. Comparative modeling is the most precise method of predicting protein structures, as it can generate models with an accuracy that is similar to that of experimental protein models [60].

BDNF has had only part of its structure experimentally determined. The available crystallographic fragments of BDNF have no structure at the position 66 and adjacent residues [17], which are located at the pro-region of the protein and are important for the release-dependent activation of BDNF [14]. Thus, we sought to develop the *in silico* complete model of BDNF and its V66M variant.

HHPred and SwissModel returned incomplete models of BDNF, whereas Rosetta, I-Tasser, RaptorX, Phyre2 and M4T returned complete models. The alignment between the modeled structures of BDNF and its crystallographic fragment (PDB ID: 1BND) by TM-align is shown in Table 1, The alignment provides two distinct values: RMSD and TM-score, which are used to evaluate the structural similarity of the submitted molecules [58]. RMSD is defined as the spatial differences between two static structures [61]. Protein size and local structural deviations are not considered in RMSD calculation. Based on this approach, the TM-align server also provides a TM-score, which is calculated from smaller portions of the structure and is normalized according to protein size, thus providing greater sensitivity in evaluating structural similarity [58]. Accurate models present TM-scores approaching 1 and RMSD < 2.0 Å [20]. Thus, the structural alignment (Table 1) indicated the generated models are accurate. Rosetta’s model (Fig 1) presented the best RMSD and TM-score values amongst the complete models and was considered the most accurate. The structural similarity between the Rosetta’s model and its template structure, the experimental fragment of BDNF (PDB ID: 1BND), is shown in Fig 2.

The Rosetta’s model had its stereochemical quality confirmed by PROCHECK and RAMPAGE (Fig 4) and its 3D-1D structural compatibility confirmed by Verify3D (Fig 3C). Moreover, the ProSa and QMEAN assessment showed that the overall quality of the Rosetta’s model is comparable to that of experimental determined protein structures (Fig 3B and 3C).

To further validate the modeled protein structure, we compared its secondary structure with the 1BND experimental fragment and with the CONCORD results (Fig 5). It indicates that the theoretical model of BDNF also shows high accuracy regarding its secondary structure. With the quality assessment and the confirmation of the secondary structure prediction, the theoretical model of BDNF was considered a high-quality model and selected for the subsequent analyses.

Structurally and functionally important residues of a protein are usually conserved along evolution. Thus, the biological importance of a residue can be correlated to its level of evolutionary conservation [49]. We used ConSurf to investigate the evolutionary conservation of the position 66 of BDNF. ConSurf estimates the evolutionary conservation of amino acids in a protein and attributes a conservation score to them [49].

Although the V66M mutation of BDNF is described as being associated with the development of psychiatric disorders [13,15] and occurs in a position that is determinant for the intracellular sorting and activity-dependent secretion of the protein [14], the ConSurf analysis classified position 66 of BDNF as variable and buried (Fig 6). This result could be related to the low sensitivity of the functional prediction algorithms used to predict the known deleterious potential of the V66M mutation on BDNF [13,15], as most are based on evolutionary information from the sequence to make predictions [24].

We also developed a complete theoretical model of the V66M variant of BDNF to further investigate the effects of V66M mutation on BDNF. The comparison between the *in silico* modeled structure of wild-type BDNF and its variant V66M (Fig 7) shows that the V66M substitution does not affect the side chain interactions of residue 66, as well as the possible hydrogen bonds formed. Interestingly, the hydrogen-bonding analysis we carried out (Table 2), pointed to differences in the occupancy of hydrogen bonds formed throughout the BDNF wild-type and its V66M variant simulations, indicating that the V66M mutation may affect the BDNF propensity for hydrogen bond formation at its pro-domain.

The impact of mutations on the structure and function of a protein can be thoroughly examined using MD simulations [62]. We then performed MD simulations of wild-type BDNF and its V66M variant to investigate the effects of the V66M mutation on BDNF [70]. The plateau of RMSD values, observed at both simulations (Fig 8), is similar between the BDNF wild-type and V66M variant, indicating that both structures fluctuate around a stable average conformation [61,63]. Moreover, the RMSD values of the V66M variant at the plateau are similar to those of the wild-type structure. The RMSF (Fig 9) and B-factor (Fig 10) analyses indicated no local flexibility alterations between the wild-type BDNF and its V66M variant. The changes observed in the Rg (Fig 13) and SASA (Fig 14) analyses suggested that the V66M mutation does not affect the surface-to-volume ratio of BDNF.

Since the average RMSD value of the entire simulations of wild-type BDNF (0.55 ± 0.04nm and 0.53 ± 0.05nm, respectively) and its variant V66M (0.58 ± 0.07nm and 0.62 ± 0.04nm, respectively) are similar to those at their plateaus, we then submitted the full MD trajectories of wild-type BDNF and its variant V66M to Bio3D for the PC analysis in order to capture their molecular motions for a longer time (200ns of entire simulation, instead of 100ns of plateau time).

The alterations observed in the PC analysis (Fig 11) indicated that the V66M mutation could affect the BDNF essential motions, particularly at its N-terminus (Fig 12). Considering that the biological functions of proteins are usually determined by their essential motions [67], such as binding to substrates and protein interactions [69], the alterations observed during the PC analysis, could be related to the functional impairment of BDNF upon V66M. Especially because the BDNF pro-domain, located at its N-terminus, is determinant for the intracellular sorting, activity-dependent secretion [14], molecular folding [77], and proteolytic cleavage of BDNF [78].

Although the RMSF and B-factor analyses suggested that the V66M mutation does not affect BDNF local flexibility, the PC analysis indicated that this mutation could affect the BDNF essential dynamics. The process of applying PC analysis to a protein trajectory is also known as essential dynamics, since the essential motions of a given protein are extracted from a set of sampled conformations [68]. Certain types of internal motions, such as binding to substrates and protein interactions, allow proteins to perform their biological functions. The aim of essential dynamics, in this sense, is to determine how these movements relate to protein function and to identify the most representative degrees of freedom. The essential dynamics allows to separate a set of internal atomic movements into two subspaces: 1) the essential subspace, which contains only a few degrees of freedom [69] usually describing relevant movements to protein function [68], such as opening, closing, and flexing; 2) and the remaining subspace, which describes small irrelevant local fluctuations [69]. Thus, the PC analysis, which describes the essential movements of a protein [68], usually better characterize its mechanical behavior [70] rather than the RMSF and B-factor analysis, which describe both relevant and irrelevant local fluctuations.

The alterations observed in the secondary structure analysis (Fig 15) suggested that the V66M mutation could affect the BDNF secondary structure at its N-terminus, especially at the BDNF first alpha-helix (pre-domain). The BDNF pre-domain leads the newly-generated protein to the endoplasmic reticulum, where it is cleaved to pro-BDNF. This constitutes a crucial step in the processing of BDNF [87]. The secondary structure analysis also pointed to little alterations at the BDNF pro-domain, which is crucial for BDNF functionality [14,77,78]. Thus, considering that the function of most proteins is guided by interactions with other molecules and, consequently, dependent on hydrogen-bonding [73] and secondary structure formation [73–75], the alterations observed in the secondary structure (Fig 15) and hydrogen-bonding analysis (Table 2) further indicate that the V66M mutation may affect BDNF functional interactions particularly at its pre and pro-domain.

The MD analyses suggested that the V66M mutation does not affect the BDNF local flexibility and surface-to-volume ratio, but could affect the BDNF essential motions, particularly at its N-terminus. The MD analyses also suggested that the V66M mutation could affect the BDNF hydrogen-bonding at its pro-domain, as well as its secondary structure at the N-terminus (pre and pro-domain). Thus, considering that hydrogen-bonding, secondary structure formation and essential dynamics are determinant for protein interactions and, consequently, protein function [69,73–75]; the alterations observed throughout the MD simulations may be associated with the functional impairment of BDNF upon this mutation [67], as well as its involvement in psychiatric disorders [8,13]. Especially because these alterations may impact BDNF effective interactions at the pre and pro-domain, which are fundamental for the processing [87], intracellular sorting, activity-dependent secretion [14], molecular folding [77], and proteolytic cleavage of BDNF [78].

## Conclusions

Computational (*in silico*) approaches are efficient and necessary to predict protein structures and study disease-associated mutations. This work provided an accurate and complete model of BDNF *in silico*. Stability and functional predictions pointed to the low accuracy of the algorithms used in predicting the known deleterious potential of the V66M mutation and showed the importance of combining more than one method to predict the effects of non-synonymous mutations. Phylogenetic analysis indicated that position 66 of BDNF is not conserved; however, it is fundamental for the intracellular sorting and activity-dependent secretion of the protein. The conservation degree of position 66 of BDNF could also be related to the low accuracy of the algorithms used in predicting the known deleterious potential of the V66M mutation, since most of them are based on evolutionary information to make predictions. Furthermore, MD analyses indicated that the V66M mutation does not affect the BDNF flexibility and surface-to-volume ratio, but affects the BDNF essential motions, hydrogen-bonding and secondary structure formation, particularly at its N-terminus. Thus, considering that these parameters are determinant for protein interactions and, consequently, protein function; the alterations observed during the MD analyses may be related to the functional impairment of BDNF upon V66M mutation, as well as its involvement in psychiatric disorders. Especially because the BDNF N-terminus, which contains its pre and pro-region, is crucial for its activity and distribution.
